# Clocks Made of Flowers

**Published:** 1861-05

**Authors:** 


### CLOCKS MADE OF FLOWERS.

The periodicity of plants in opening and closing their blossoms, has enabled botanists to form floral dials or clocks, by means of which the different hours of the day may be ascertained.

At 3 o'clock A. M., the Goatsbeard blossom opens.
At 4 o'clock the Dandelion.
At 5 o'clock the Hawk's-beard, (Crepis teetorium.)
At 6 o'clock the Vipers-grass, (Scarzonera.)
At 7 oclock, flowers of the common Lettuce open.
At 8 o'clock, Venus' looking-glass, (Specularie specu.)
At 9 o'clock, Creeping mouse-ear hawk-weed.
At 10 o'clock, the purple savin, (Juniperus sabina.)
At 11 o'clock, the Star of Bethlehem.

No plant by its flowering distinctly marks mid-day, although many varieties of fig-trees do blossom about that time.

At 1 P. M., the Succory (Chicorium,) opens.
At 2 P. M., the Squill Hyacinth.
At 3 P. M., the common Marigold, (not reliable.)
At 4 P. M., the Four-o'clock.
At 5 P. M., the Flower-of-the-wall (Hieracum murarum.
At 6 P. M., Evening Primrose.
At 7 P. M., the Night-blooming Cereus, (Noctiflora-)
At 8 P. M., Marvel of Peru, (Mirabilis jalapa) uncertain.
At 9 P. M., the Mournful Geranium, (Geranium trieste)

Of course, from various causes, these fair visitors are not always punctual to the minute—yet, "a plant accustomed to flower in daylight at a certain time, will continue to expand its flowers at the wonted period, even when kept in a dark room. Decandolle made a series of experiments in the flowering of plants kept in darkness, and in a cellar lighted by lamps. He found that the law of periodicity continued for some time to operate, and that in artificial light, some flowers opened, while others, such as species of Convolvulus, still followed the clock hours in their opening and closing.—*Working Farmer.*

### TOO GOOD TO LIVE LONG.

Dr. Hall somewhat widely known through his *Journal of Health*, publishes another monthly, which, from its excellence, we fear will not flourish in these degenerate times. He says of it:

"While it is not, professedly, a religious publication, it never by any chance contains a sentence, a line, or a word adverse to the Bible, to religion, or the Sabbath-day; nor a sentiment contrary to what is usually received by the friends of evangelical Christianity."—*Herald of Progress.*

